# miR-20b-5p, TGFBR2, and E2F1 Form a Regulatory Loop to Participate in Epithelial to Mesenchymal Transition in Prostate Cancer

**DOI:** 10.3389/fonc.2019.01535

**Published:** 2020-01-15

**Authors:** Jin-Chun Qi, Zhan Yang, Yan-Ping Zhang, Bao-Sai Lu, Yue-Wei Yin, Kai-Long Liu, Wen-Yong Xue, Chang-Bao Qu, Wei Li

**Affiliations:** Department of Urology, The Second Hospital of Hebei Medical University, Shijiazhuang, China

**Keywords:** prostate cancer, EMT, TGFBR2, E2F1, miR-20b-5p, TGF-β1

## Abstract

The transcription factor E2F1 regulates the expression of the miR-20b-5p precursor and is involved in epithelial-to-mesenchymal transition (EMT). Transforming growth factor-β1 (TGF-β1) induces EMT in prostate cancer (PCa) by binding to TGF-beta receptor 2 (TGFBR2) to activate TGF-β signaling. However, the relationship between TGFBR2, E2F1, and miR-20b-5p in the modulation of EMT in PCa cells remains unknown. In this study, we found that the level of miR-20b-5p expression was significantly lower in PC3 and DU145 cells than that in prostate epithelial (RWPE-1) cells, and TGF-β1 treatment further down-regulated miR-20b-5p expression in these two cell lines. Functional studies showed that miR-20b-5p suppressed TGF-β1-induced migration and invasion of PC3 and DU145 cells by up-regulating E-cadherin and down-regulating vimentin, leading to TGF-β1-induced inhibition of EMT. Using gain and loss of function experiments, it was shown that E2F1 mediated TGF-β1 regulation of miR-20b-5p expression. Further, a luciferase activity assay showed that TGFBR2 was a direct target of miR-20b-5p in PCa cells. These results suggest that miR-20b-5p, TGFBR2, and E2F1 form a regulatory loop to modulate EMT induced by TGF-β1. A novel regulatory mechanism underlying the miR-20b-5p/TGFBR2/E2F1 axis is involved in TGF-β1-induced EMT of PCa cells, and miR-20b-5p may be a potential therapeutic target for PCa.

## Introduction

Prostate cancer (PCa) is the second leading cause of cancer-related death among males in both developed and less developed countries, including China (1, 2). Patients with PCa predominantly die from metastatic disease when the cancer becomes resistant to androgen deprivation therapy, which is termed castration-resistant PCa (CRPC) (3). Epithelial-to-mesenchymal transition (EMT) is a prerequisite for cancer invasion into the surrounding tissue (4–6). EMT is closely associated with CRPC. However, the molecular mechanisms by which EMT is modulated by different regulators remain largely unclear.

Numerous microRNAs (miRNAs) are abnormally expressed in human tumors, contributing to tumor metastasis or the inhibition of metastasis through modulating EMT (7–10). For example, miR-146a is down-regulated in PCa, and increased miR-146a expression by 5-Aza-2′-deoxycytidine correlates with the delayed progression of CRPC (10). miR-30 inhibits transforming growth factor-β1 (TGF-β1)-induced EMT in hepatocytes by targeting Snail1 (11). Previous studies have shown that miR-20b-5p functions as a tumor suppressor in renal cell carcinoma and bladder cancer by regulating cellular proliferation and migration (12, 13). Recent studies have shown that miR-20b-5p plays an important regulatory role in tumor progression. For example, miR-20b-5p regulates endometrial cancer formation (14), promotes NSCLC cell proliferation and migration (15), and regulates stem cell-like properties in human colorectal cancer cells (16). However, it is unclear whether miR-20b-5p modulates EMT in PCa and how its expression is regulated in PCa cells.

TGF-β1 is a principal extracellular inducer of EMT (17). It is known that the tumor-suppressive effects of TGF-β1 are dependent on both Smad3 and the transcription factor E2F1 (18). A recent study showed that the E2F1-miR-20a-5p/20b-5p autoregulatory feedback loop is involved in myoblast proliferation and differentiation (19). The multifunctional cytokine TGF-β1 orchestrates an intricate signaling network to modulate tumorigenesis and progression (20, 21). TGF-β1 exerts its tumor-suppressive role by inducing cell-cycle arrest and apoptosis (22). Nevertheless, TGF-β1 also promotes tumor progression through enhancing proliferation, migration, and invasion, in part by its ability to induce epithelial-mesenchymal transition (EMT) (23, 24). However, it is not known whether TGF-β1-activated TGFBR2/Smad signaling regulates the E2F1-miR-20b-5p autoregulatory feedback loop and contributes to TGF-β-induced EMT pathogenesis in human PCa, and the mechanisms involved are also unclear.

This study investigated whether and how TGF-β1 regulates EMT in PCa cells through modulating the TGFBR2-E2F1-miR-20b-5p regulatory loop. Our results confirmed that miR-20b-5p targets and down-regulates TGFBR2, which in turn affects Smad2 activation and E2F1 expression, leading to dysregulation of miR-20b-5p expression and contributing to TGF-β-induced EMT in human PCa.

## Materials and Methods

### Cell Culture and Treatment

PC3, LNCaP, DU145, VCaP, and 22RV1 cells were purchased from ATCC and grown in RPMI 1640 medium (Gibco). All media contained 10% fetal bovine serum (FBS), penicillin (100 units/mL), and streptomycin (100 μg/mL). RWPE-1 cells (CRL-11609, ATCC) were maintained in K-SMF medium (Life Technologies) supplemented with 5 ng/mL epidermal growth factor and 50 μg/mL bovine pituitary extract. Cultures were incubated in a humidified environment at 5% CO_2_ at 37 °C. All cells were transfected using Lipofectamine 2000 (Life Technologies) according to the manufacturer's instructions. To stimulate cells, 2 ng/mL TGF-β1 (R&D Systems) was used, using the method described previously (25).

### Cell Migration and Invasion

To study cell invasion and migration, transwell membranes were coated with Matrigel (Sigma-Aldrich), or left uncoated, prior to plating cells. PC3 or DU145 cells of 3 × 10^4^ cells/well were seeded on the upper chambers with serum-free medium. The medium containing 10% FBS was added to the lower chambers. After culturing at 37 °C and 5% CO_2_ for 24 h, cells with higher migration and invasion capacity on the upper side of the chamber were migrated into the lower chamber. The migration and invasion rate was determined by digital image processing software of ImageJ.

### Vector Construction and Luciferase Activity Assay

Plasmids were constructed using restriction-enzyme digestion and one-step cloning (C112-02; Vazyme Biotech Co.) or other recombinant methods. The 3′ untranslated region (UTR) sequences of TGFBR2 containing wild-type (WT) or mutant forms of the miR-20b-5p-binding site were inserted into the pmir-GLO Dual-Luciferase miRNA Target Expression Vector (Promega Corp.). Overexpression of E2F1 was performed using a pCMVHA-E2F1 vector (#24225, Addgene). The luciferase activity was measured using a Dual-Glo Luciferase Assay System (Promega Corp.) with a Flash and Glow reader (LB955) 24 h after transfection. The specific target activity was expressed as the relative activity ratio of firefly luciferase to Renilla luciferase.

### Western Blot Analysis

Proteins from cultured cells were prepared with a lysis buffer using the method previously described (26). Equal amounts of proteins were separated on SDS-PAGE and electro-transferred to a PVDF membrane (Millipore). Membranes were blocked with 5% milk in TTBS for 2 h at room temperature and incubated with primary antibodies overnight at 4°C. The antibodies used were as follows: anti-E-cadherin (20874-1-AP), anti-vimentin (10366-1-AP), anti-β-actin (sc-47778), anti-E2F1 (ab94888), anti-ZO-1 (66452-1-Ig), anti-ZO-1 (66452-1-Ig), anti-N-cadherin (22018-1-AP), anti-Smad2 (sc-5339), and anti-phospho-Smad2 (sc-8828). The membranes were then incubated with the HRP-conjugated secondary antibody (Rockland) for 1 h at room temperature. The blots were treated with the Immobilon™ Western (Millipore) and detected by enhanced chemiluminescence Fuazon Fx (Vilber Lourmat). Images were captured and processed with Fusion Capt Advance Fx5 software (Vilber Lourmat). All experiments were replicated three times.

### Isolation of RNA and PCR

Cultured cells were lysed using the QIAzol Lysis Reagent (Catalog no.79306). Total RNA was extracted from the sample according to the manufacturer's instructions (Catalog no.217004). The quality of the RNA was determined using a Nanodrop 2000 (Thermo). For miRNA, reverse transcription and qRT-PCR were performed using the miRNA Detection Kit (Genepharma) and internal control U6, according to the manufacturer's instructions. For large mRNA, cDNA was synthesized using an M-MLV First Strand Kit (Life Technologies) with random hexamer primers. qRT-PCR of mRNAs was performed using Platinum SYBR Green qPCR Super Mix UDG Kit (Invitrogen), and real-time PCR experiments were carried on a ABI 7500 FAST system (Life Technologies). The relative amount of transcripts was normalized with GAPDH and calculated using the 2^−ΔΔ^Ct formula, as previously described (27). For RT-PCR of mRNAs, 5 μL of 1:5 diluted cDNA was amplified in a 25 μL PCR reaction using the KOD Xtreme^TM^ HotStart Polymerase Kit (71975-3, Novagen) with primers of E2F1 forward: GGA GGC TGG ACC TGG AAA CTG ACC; E2F1 reverse: CTC AAG GAC GTT GGT GAT GTC ATA GATG; TGFBR2 forward: GGG AGT TGC CAT ATC TGT CAT CAT CAT CTTC; TGFBR2 reverse: GTT CTG CTT CAG CTT GGC CTT ATA GACC; GAPDH forward: AAG GTG AAG GTC GGA GTC; and GAPDH reverse: GAT TTT GGA GGG ATC TCG.

### Small Interfering RNA Transfection

Small interfering RNAs (siRNAs) targeting human TGFBR2 (si-TGFBR2) and E2F1 (si-E2F1) were designed by BioCaring Biotechology (Shijiazhuang, China) and synthesized by Gene Pharma (Shanghai, China). The siRNA sequences were as follows: siTGFBR2-F: GAG GCC CAG AAA GAU GAA ATT; siTGFBR2-R: UUU CAU CUU UCU GGG CCU CTT; siE2F1-F: CUG CAG AGC AGA UGG UUA UTT; and siE2F1-R: AUA ACC AUC UGC UCU GCA GTT. Non-specific siRNA (si-Control), miR-20b-5p mimic, and its antagomir (inhibitor) were purchased from Gene Pharma (Shanghai). Transfection was performed using Lipofectamine 2000 following the manufacturer's instructions. Cells were then harvested and lysed for western blotting or PCR.

### Xenograft Animal Model

All animal studies were approved by the Institutional Animal Care and Use Committee of Hebei Medical University (approval ID: HebMU 20080026), and all efforts were made to minimize suffering. A xenograft model was performed as previously described (27). Male BALB/c nude mice (*n* = 12) at 4–6 weeks of age (18–22 g) were purchased from Vital River Laboratory Animal Technology Co., Ltd. (Beijing, China). LV-Ctl- or LV-miR-20b-5p-infected PC3 cells of 5 × 10^6^ were harvested by trypsinization and resuspended in 0.2 mL PBS mixed with 50% Matrigel (Collaborative Research Inc.). The suspension was injected subcutaneously into the right dorsal flanks. The length and width of mouse tumors were measured twice a week with calipers. The formula volume = ([length × width2]/2) was used to calculate tumor volume. At the end of the experiment, the mice were euthanized by carbon dioxide asphyxiation.

### Target Prediction

Potential target genes of miR-20b-5p were identified using miRNA target prediction algorithms, including miRanda (www.microrna.org) and RNAhybrid (http://bibiserv.techfak.uni-bielefeld.de/rnahybrid/submission.html) (28, 29).

## Results

### TGF-β1 Depresses the Expression of miR-20b-5p in PCa Cells

**Figure 1:**
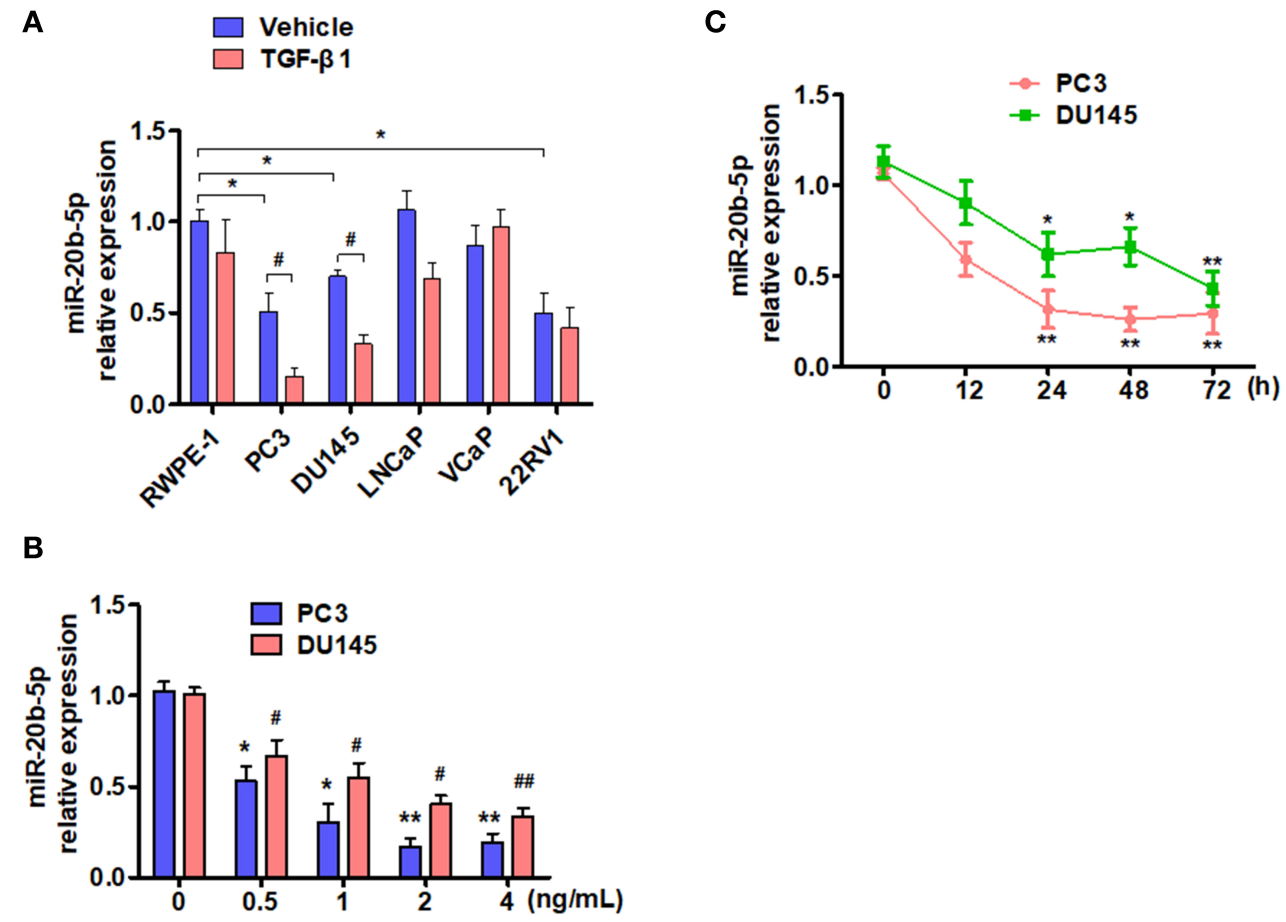
TGF-β1 depresses the expression of miR-20b-5p in PCa cells. **(A)** Different PCa cell lines were treated with or without TGF-β1 for 24 h, and the expression of miR-20b-5p was analyzed by qRT-PCR. *n* = 3, *P < 0.05 vs. RWPE-1 vehicle; ^#^*P* < 0.05 vs. their corresponding vehicle. **(B)** PC3 and DU145 cells were treated with different concentrations of TGF-β1, and miR-20b-5p expression was measured by qRT-PCR at 0, 0.5, 1, 2, and 4 ng/mL after TGF-β1 treatment. *P < 0.05 vs. PC3 with 0 ng/mL TGF-β1; ^#^*P* < 0.05, ^*##*^*P* < 0.01 vs. DU145 with 0 ng/mL TGF-β1. **(C)** PC3 and DU145 cells were treated with 2 ng/mL of TGF-β1 for different times, miR-20b-5p expression was measured by qRT-PCR at 0, 12, 24, 48, and 72 h after TGF-β1 treatment. MicroRNA expression values were rescaled relative to the blank control. *P < 0.05, ^**^P < 0.01 vs. 0 h.

TGF-β is known to depress the transcriptional activity of E2F-1, which is down-regulated by miR-20b-5p (19, 30). We sought to determine whether the expression of miR-20b-5p in PCa cell lines is affected by TGF-β. To do so, we treated five different PCa cell lines with 2 ng/mL TGF-β1, as described previously (25), and evaluated miR-20b-5p expression by qRT-PCR. As shown in Figure 1A, the basal expression of miR-20b-5p was lower in PC3, DU145, and 22RV1 cells than that in other cell lines tested. miR-20b-5p was significantly down-regulated in TGF-β1-treated PC3 and DU145 cells (*P* < 0.05), whereas TGF-β1 did not significantly affect the level of miR-20b-5p expression in LNCaP, VCaP, and 22RV1 cells. We also examined the inhibitory effect of TGF-β1 on miR-20b-5p expression, and confirmed that treating PC-3 and DU145 cells with different concentrations of TGF-β1 led to a dose-dependent decrease in miR-20b-5p level (Figure 1B). To confirm that the expression of miR-20b-5p in PC3 and DU145 cells was regulated by TGF-β1, we treated the cells with 2 ng/mL TGF-β1 for different lengths of time and found that the expression levels of miR-20b-5p were gradually reduced as the time after treatment with TGF-β1 increased to 0, 12, 24, 48, and 72 h, with a significant reduction in the miR-20b-5p level noted at 24 h after TGF-β1 treatment (Figure 1C). We used qRT-PCR to detect miR-20b-5p expression in PCa tissues and benign prostatic hyperplasia (BPH). The results showed that the miR-20b-5p level was significantly decreased in PC tissues from 30 patients, compared with that in tissues from BPH patients (Supplementary Figure 1). These findings suggest that miR-20b-5p expression was down-regulated in PCa tissues, as well as in both PC3 and DU145 cells, and that TGF-β1 treatment further decreased miR-20b-5p levels in a time-dependent manner.

### miR-20b-5p Suppresses TGF-β1-Induced EMT in PC3 and DU145 Cells Through Regulating EMT-Related Gene Expression

**Figure 2:**
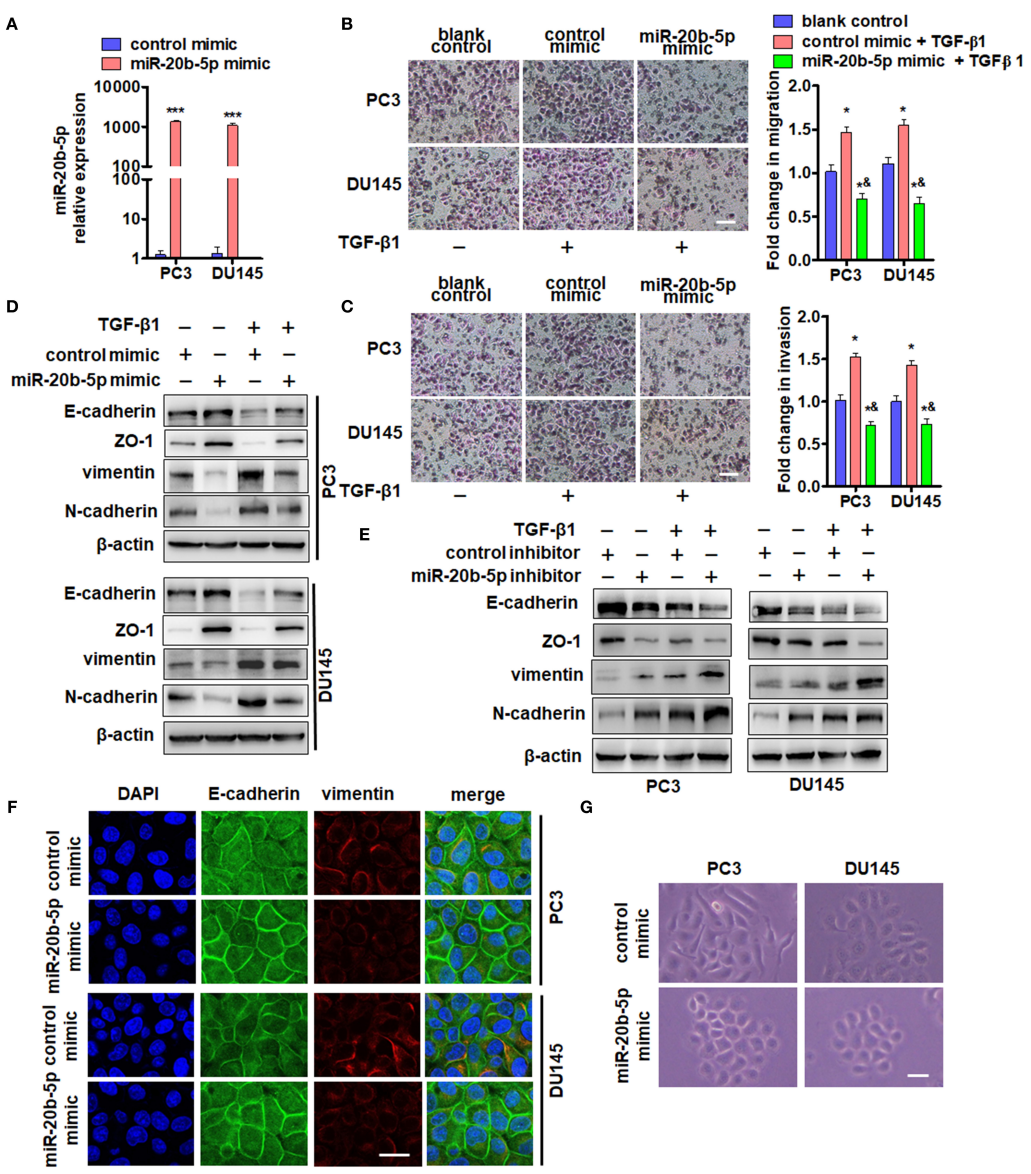
miR-20b-5p suppresses migration and invasion of PC3 and DU145 cells by regulating EMT-related gene expression. **(A)** qRT-PCR detected the expression of miR-20b-5p in PC3 and DU145 cells transfected with a miR-20b-5p mimic or control mimic for 24 h. ***P < 0.001 vs. control mimic. Migration **(B)** and invasion **(C)** assays for PC3 and DU145 cells transfected with a miR-20b-5p mimic and treated with 2 ng/mL TGF-β1. Representative images were taken at ×200 magnification; the numbers of migrated cells on the lower surface of the inserts were quantified in four random fields from each treatment group. *P < 0.05, ***P < 0.001 vs. blank control; ^&^*P* < 0.001 vs. control mimic + TGF-β1; bar = 200 μm. **(D)** PC3 and DU145 cells were transfected with a miR-20b-5p or control mimic and then treated with or without TGF-β1. Western blot analysis detected E-cadherin, ZO-1, vimentin, and N-cadherin. **(E)** PC3 and DU145 cells were transfected with miR-20b-5p or control inhibitor and then treated with or without TGF-β1; E-cadherin, ZO-1, vimentin, and N-cadherin expression was detected by western blotting. All experiments were performed in triplicate. **(F)** Immunofluorescence staining detected the expression of E-cadherin (green) and vimentin (red) in miR-20b-5p mimic-transfected PC3 and DU145 cells. Nuclei were counterstained with DAPI (blue). Scale bars = 25 μm. **(G)** Phase-contrast microscope observed cell morphology of PC3 and DU145 with miR-20b-5p mimic transfection. Scale bars = 25 μm.

In the primary PCa tumor, epithelial cells undergo EMT and subsequently become invasive and metastatic (31). We investigated whether miR-20b-5p impacted the migration and invasion of PC3 and DU145 cells by regulating EMT-related gene expression. Our results confirmed that the miR-20b-5p increased over 1000-fold in miR-20b-5p mimic-transfected PC3 and DU145 cells (Figure 2A). Subsequently, we examined the effects of miR-20b-5p overexpression on cell migration and invasion using Transwell and Matrigel invasion assays. As shown in Figures 2B,C and Supplementary Figure 2, PC3 and DU145 cells that passed through the membrane or invaded the Matrigel were significantly reduced upon the overexpression of miR-20b-5p, regardless of the presence or absence of TGF-β1. Next, we examined the effect of miR-20b-5p and TGF-β1 on the expression of epithelial marker E-cadherin, ZO-1, and the mesenchymal markers vimentin and N-cadherin. The transfection of miR-20b-5p mimics significantly increased E-cadherin and ZO-1 expression but decreased the vimentin and N-cadherin level (Figure 2D, lane 2 vs. lane 1). TGF-β1 treatment partly counteracted the effect of miR-20b-5p overexpression (Figure 2D and Supplementary Figure 3A). In contrast, depletion of miR-20b-5p by its antagomir markedly reduced E-cadherin and Zo-1, and it elevated the vimentin and N-cadherin level when compared with control mimics (Figure 2E, lane 2 vs. lane 1). TGF-β1 further enhanced the effect of the miR-20b-5p inhibitor (Figure 2E and Supplementary Figure 3B). Immunofluorescence staining of miR-20b-5p mimic-transfected PC3 and DU145 cells for E-cadherin and vimentin was performed. The results showed that miR-20b-5p mimic transfection led to an obvious increase in E-cadherin in the cell membrane and decreased expression of vimentin (Figure 2F). Similarly, miR-20b-5p mimic-transfected PC3 and DU145 cells to change to an epithelial phenotype (Figure 2G), whereas TGF-β1 treatment reversed this cell morphology (Supplementary Figure 4). These data suggest that miR-20b-5p suppresses TGF-β1-induced migration and invasion of PC3 and DU145 cells by up-regulating E-cadherin and ZO-1 and down-regulating vimentin and N-cadherin.

### E2F1 Acts as a Mediator of TGF-β1 Regulation of miR-20b-5p Expression

**Figure 3:**
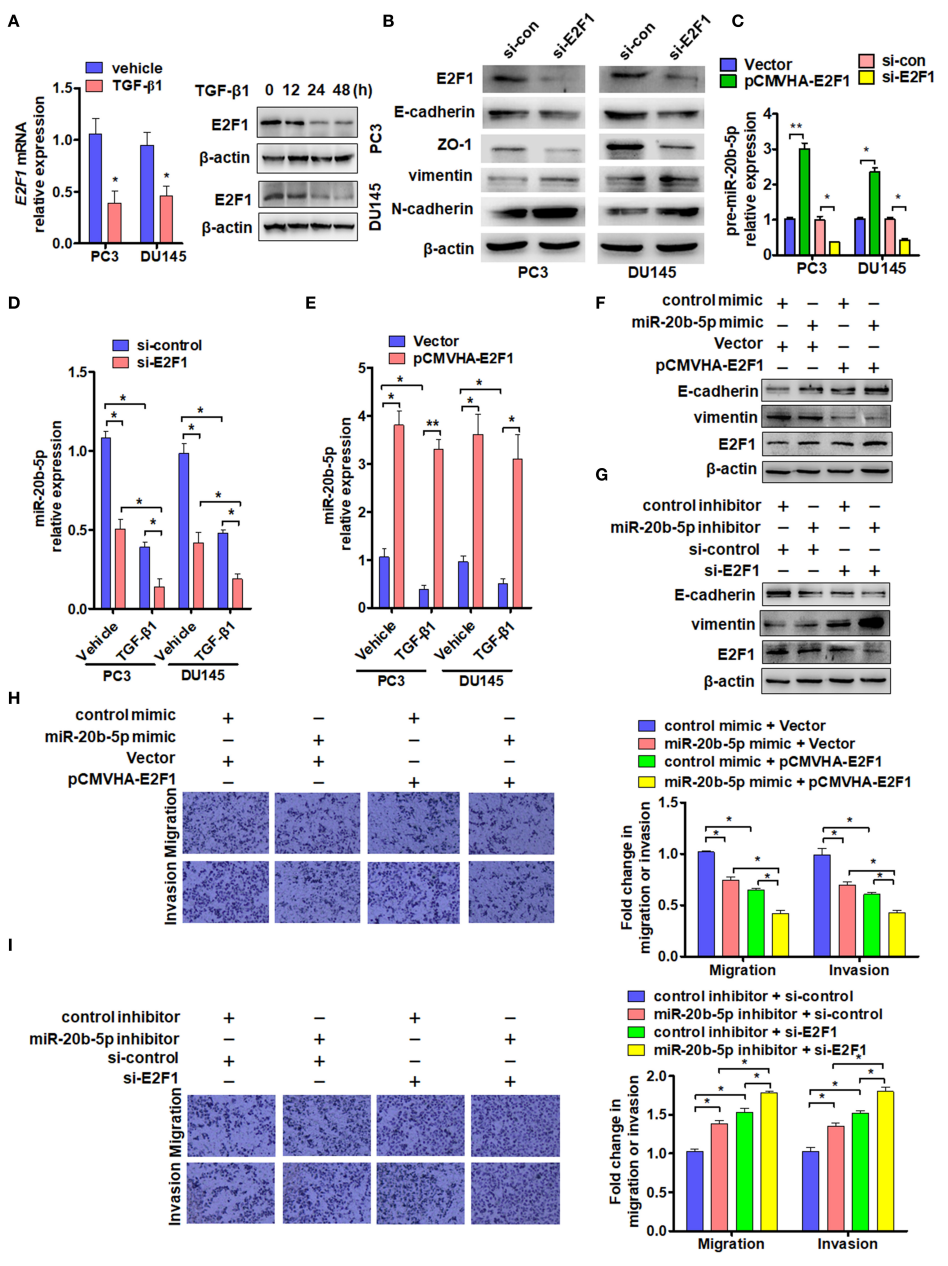
E2F1 mediates TGF-β1 regulation of miR-20b-5p expression. **(A)** PC3 and DU145 cells were treated with TGF-β1 (2 ng/mL) for 24 h, and E2F1 mRNA was detected by qRT-PCR. *P < 0.05 vs. vehicle (left panel). Western blot analysis detected E2F1 protein in PC3 and DU145 cells treated with 2 ng/mL of TGF-β1 for the indicated times (right panel). **(B)** Western blot detected EMT relative protein in PC3 and DU145 cells transfected with si-E2F1 or control siRNA of TGF-β1. **(C)** PC3 and DU145 cells were transfected with indicated RNA and vectors, and qRT-PCR detected the expression of pre-miR-20b-5p. *P < 0.05, **P < 0.01 vs. indicated control. **(D)** Expression of miR-20b-5p was measured by qRT-PCR in PC3 and DU145 cells transfected with si-E2F1 or si-control and treated with or without TGF-β1. *P < 0.05 vs. corresponding control. **(E)** PC3 and DU145 cells were transfected with pCMVHA-E2F1 or an empty vector and treated with or without TGF-β1; qRT-PCR detected the expression of miR-20b-5p. *P < 0.05, **P < 0.01 vs. corresponding control. **(F)** PC3 cells were transfected with pCMVHA-E2F1 and a miR-20b-5p mimic or control mimic; E-cadherin, vimentin, and E2F1 expressions were determined by western blotting. **(G)** PC3 cells were transfected with si-E2F1 and a miR-20b-5p inhibitor or control inhibitor; western blot analysis detected the expression of E-cadherin, vimentin, and E2F1. All experiments were performed in triplicate. **(H,I)** cell migration and invasion of PC3 cells treatment as **(F,G)** were evaluated by transwell assay. *P < 0.05, **P < 0.01 vs. indicated control.

As transcription factor E2F1 is known to regulate the cell cycle, proliferation, apoptosis, and differentiation and is involved in tumor invasion and metastasis (30, 32), we sought to determine the effect of TGF-β1 on the expression of E2F1. As expected, exposure of PC3 and DU145 cells to TGF-β1 significantly reduced the expression of E2F1 mRNA 24 h after TGF-β1 treatment and also decreased E2F1 protein level in a time-dependent manner. E2F1 expression was significantly reduced at 24 h and reached its lowest level at 48 h after treatment with TGF-β1 (Figure 3A). Previous studies have found that E2F1 regulates the expression of EMT marker genes (19). As expected, knockdown of E2F1 in PC3 or DU145 cells significantly reduced E-cadherin and Zo-1 but elevated vimentin and N-cadherin expression (Figure 3B). Previous studies have shown that E2F1 can directly regulate the expression of miR-17~92 and miR-106a~363 gene clusters (19). Therefore, we sought to determine whether E2F1 could regulate miR-20b-5p expression directly in PCa cells. The qRT-PCR showed that overexpression of E2F1 promoted pre-miR-20b-5p expression, whereas knockdown of E2F1 inhibited pre-miR-20b-5p expression in both cell lines (Figure 3C). We also found that TGF-β1 treatment decreased the miR-20b-5p level in E2F1-depleted cells (Figure 3D). We then overexpressed E2F1 with E2F1 expression vector pCMVHA-E2F1 and found that the overexpression of E2F1 in PC3 or DU145 cells markedly increased the miR-20b-5p level, whereas TGF-β1 no longer reduced the expression of miR-20b-5p in the E2F1-overexpressing cells (Figure 3E and Supplementary Figure 5A). These data suggest that E2F1 mediates TGF-β1 regulation of miR-20b-5p expression.

In this study, we evaluated the role of E2F1 in miR-20b-5p-regulated EMT. As shown in Figure 3F and Supplementary Figure 5B, the overexpression of E2F1 increased E-cadherin and reduced vimentin protein levels in miR-20b-5p mimic-transfected PC3 cells (lane 3 vs. lane 4), inhibiting the process of EMT through up-regulating E-cadherin and down-regulating vimentin. In contrast, knockdown of E2F1 combined with miR-20b-5p knockdown could reverse the expression level of E-cadherin and vimentin in PC3 cells (Figure 3G). miR-20b-5p-mimic-transfected PC3 cells significantly inhibited cell migration and invasion, compared with those transfected with the control mimic, and the inhibitory effect could be enhanced in E2F1-overexpressed PC3 cells (Figure 3H). Conversely, a miR-20b-5p inhibitor promoted cell migration and invasion compared with cells transfected with the control inhibitor, and this promotion effect could be intensified in E2F1-depleted PC3 cells (Figure 3I). These results indicate that E2F1 regulates miR-20b-5p expression, which in turn affects EMT-related gene expression.

### TGFBR2 Is a Direct Target of miR-20b-5p in PC3 and DU145 Cells

**Figure 4:**
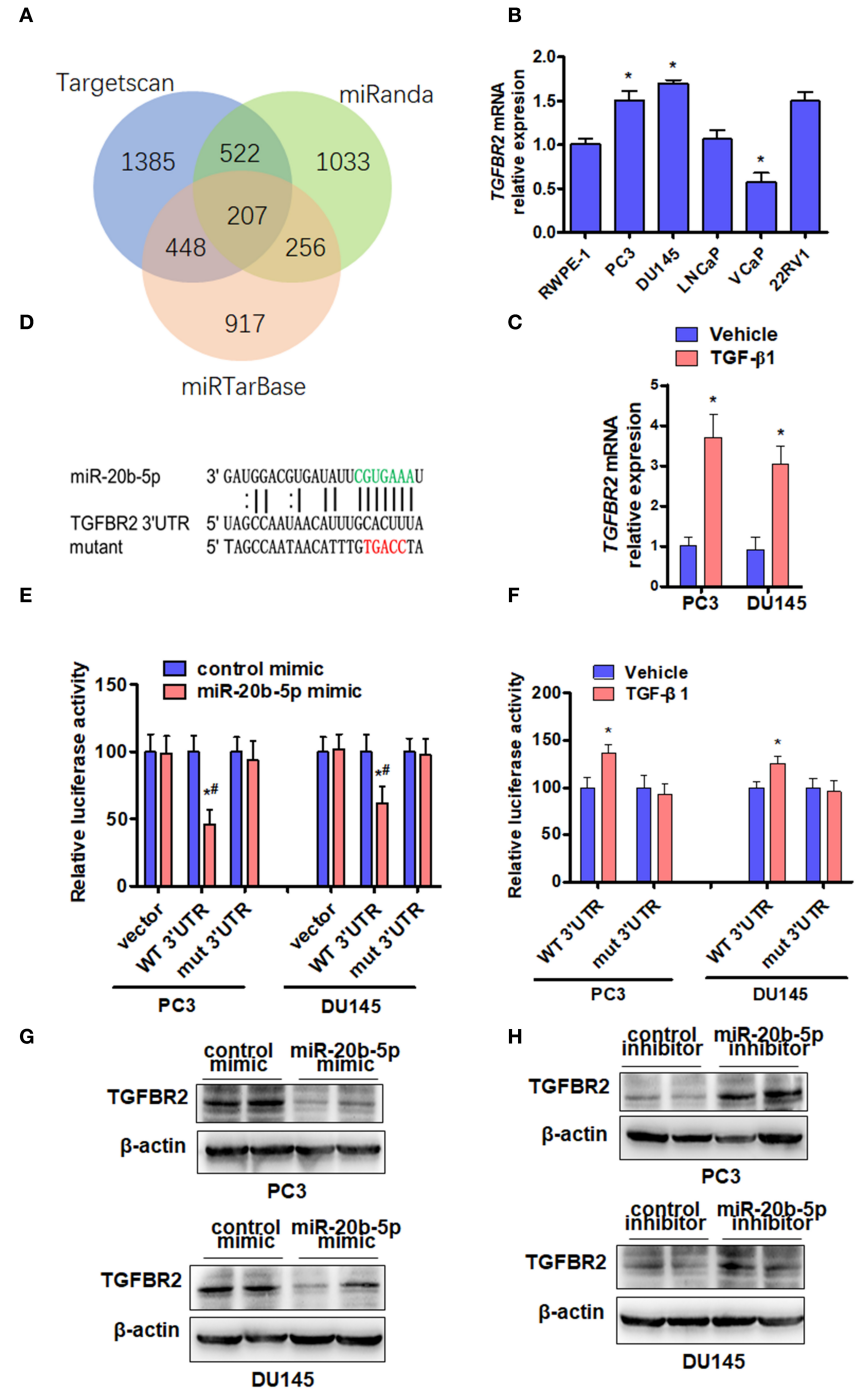
TGFBR2 is a direct target of miR-20b-5p. **(A)** Venn diagram hybrid of potential target genes of miR-20b-5p from three different target prediction programs. **(B)** The expression of TGFBR2 mRNA was detected by qRT-PCR in the different PCa cell lines. *n* = 3, *P < 0.05 vs. RWPE-1. **(C)** TGFBR2 mRNA was detected by qRT-PCR in TGF-β1-treated PC3 and DU145 cells for 48 h. *P < 0.05 vs. vehicle. **(D)** The seed sequence of miR-20b-5p that binds the 3′-UTR of the TGFBR2 mRNA (green) and the mutated site of TGFBR2 3′-UTR (red) are shown. **(E)** PC3 and DU145 cells were co-transfected with miR-20b-5p mimics or control mimics and WT (WT 3′-UTR) or mutated (mut 3′-UTR) TGFBR2 3′-UTR-luciferase reporter, luciferase activity was measured using a Dual-Glo Luciferase Assay System (*n* = 3 independent experiments per group). *P < 0.05 vs. control mimic + WT 3′-UTR; ^#^*P* < 0.05 vs. miR-20b-5p mimic + empty vector. **(F)** PC3 and DU145 cells were transfected with TGFBR2 3′-UTR-luciferase reporter (WT 3′-UTR, mut 3′-UTR) and treated with or without TGF-β1; luciferase activity was measured as in **(E)**. *P < 0.05 vs. vehicle + WT 3′-UTR. PC3 and DU145 cells were transfected with a miR-20b-5p mimic **(G)** or miR-20b-5p inhibitor **(H)**, and western blotting detected the protein level of TGFBR2. All experiments were performed in triplicate.

To study the mechanisms by which miR-20b-5p inhibits EMT, we used miRNA target prediction algorithms, including TargetScan, miRTarbase, and miRanda, to identify target genes of miR-20b-5p (Figure 4A). Among all the predicted targets of miR-20b-5p, we decided to focus on TGFBR2 due to the important position it occupies in the TGF-β1 signaling pathway. In addition, parts of these targets are involved in biological adhesion in the function of biological processes (Supplementary Figure 6). We then determined the expression of endogenous TGFBR2 in various PCa cell lines. The results showed that endogenous TGFBR2 in various PCa cells was significantly higher than that in prostate epithelial cells (RWPE-1) (Figure 4B). We then sought to determine whether the expression of TGFBR2 was regulated by TGF-β1. qRT-PCR analysis showed that treating PC3 and DU145 cells with TGF-β1 significantly increased the expression of TGFBR2 in PC3 and DU145 cell lines (Figure 4C). We constructed luciferase reporter containing WT or mutant (mut) 3′-UTR of TGFBR2 (Figure 4D) directly fused to the downstream of the Firefly luciferase gene and co-transfected these reporters with miR-20b-5p mimics into PC3 and DU145 cells. As expected, the miR-20b-5p mimic significantly decreased the luciferase activity mediated by WT TGFBR2 3′-UTR but had no effect on the mutant 3′-UTR vector (Figure 4E), suggesting the specificity of the binding of miR-20b-5p to the TGFBR2 3′-UTR. Since TGF-β1 reduced the expression of miR-20b-5p in PCa cells (Figure 1B), we tested the effect of TGF-β1 on luciferase activity mediated by TGFBR2 3′-UTR in PC3 and DU145 cells. As shown in Figure 4F, TGF-β1 significantly increased luciferase activity mediated by WT TGFBR2 3′-UTR, but not the mutant 3′-UTR, indicating that miR-20b-5p is down-regulated by TGF-β1. To confirm these observations, we transfected PC3 and DU145 cells with miR-20b-5p or a control mimic and observed that the miR-20b-5p mimic dramatically depressed TGFBR2 expression compared with the control mimic (Figure 4G and Supplementary Figure 7A). Conversely, transfection with the miR-20b-5p inhibitor markedly increased TGFBR2 expression (Figure 4H and Supplementary Figure 7B). Together, these data strongly support our notion that miR-20b-5p negatively regulates TGFBR2 expression by targeting its 3′-UTR in PCa cells.

### miR-20b-5p, TGFBR2, and E2F1 Form a Regulatory Loop to Regulate EMT Induced by TGF-β1

**Figure 5:**
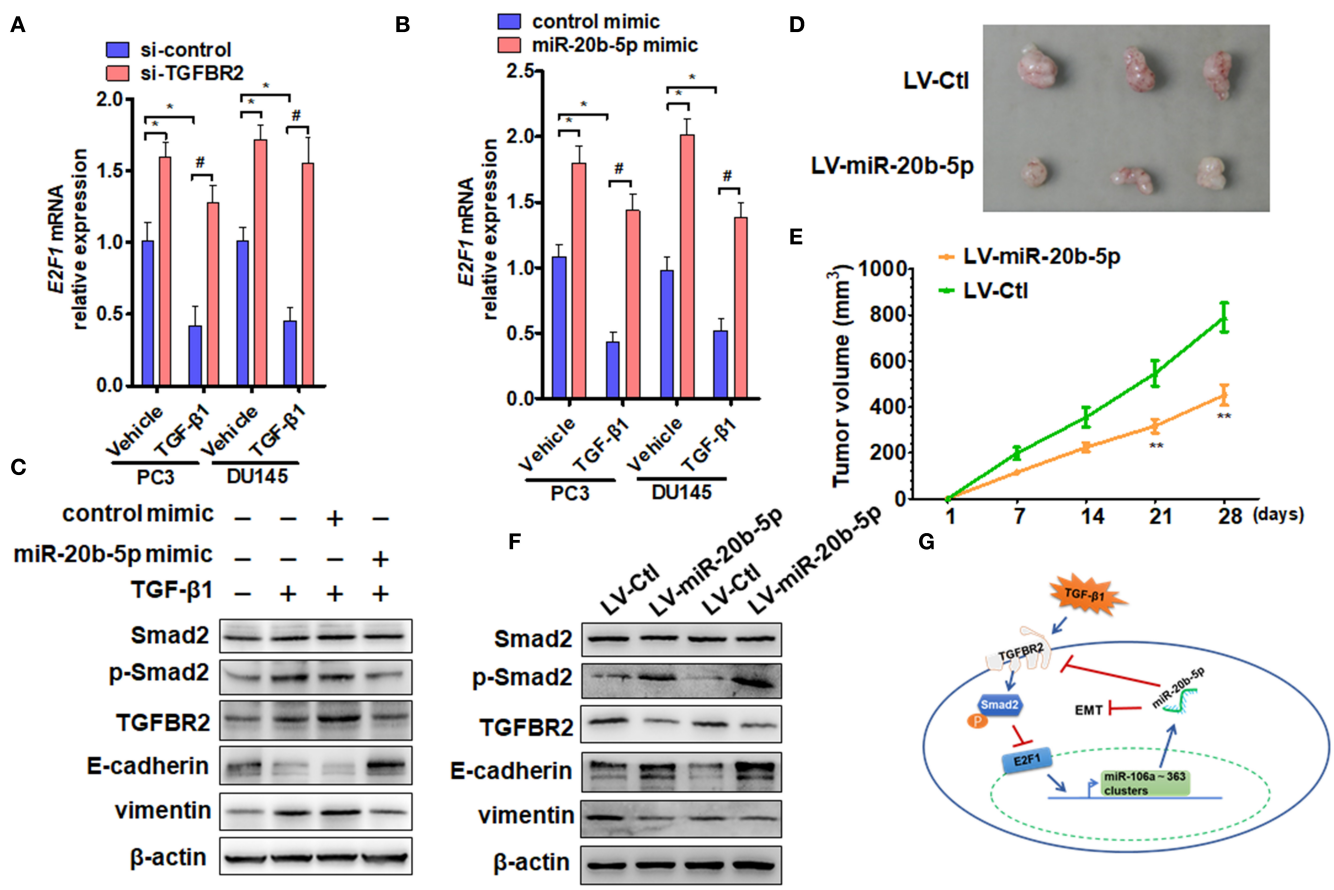
miR-20b-5p, TGFBR2, and E2F1 form a regulatory loop to participate in EMT. **(A)** PC3 and DU145 cells were transfected with si-TGFBR2 or si-control and treated with or without TGF-β1; E2F1 mRNA was determined by qRT-PCR. *P < 0.05 vs. vehicle + si-control; ^#^*P* < 0.05 vs. TGF-β1 + si-control. **(B)** qRT-PCR detected E2F1 mRNA expression in cells transfected with a miR-20b-5p mimic or control mimic and then treated with or without TGF-β1. *P < 0.05 vs. vehicle + control mimic; ^#^*P* < 0.05 vs. TGF-β1 + control mimic. **(C)** Western blot analysis detected total Smad2, phosphorylated Smad2, TGFBR2, E-cadherin, and vimentin in protein lysates from PC3 cells transfected with miR-20b-5p or a control mimic and treated with or without TGF-β1. All experiments were performed in three independent experiments. **(D)** Representative tumor sizes in each group of mice with PC3 cells engineered to stably express miR-20b-5p (LV-miR-20b-5p) or negative control (LV-Ctl). **(E)** Tumor volumes were monitored by direct measurement with calipers and calculated by the formula: (length × width2)/2. **P < 0.01 vs. LV-Ctl; **(F)** western blot analysis detected total Smad2, phosphorylated Smad2, TGFBR2, E-cadherin, and vimentin in protein lysates from xenograft tissues. **(G)** Proposed model for a feedback loop formation of TGFBR2/E2F1/miR-20b-5p in TGF-β1-regulating cell EMT.

To confirm whether TGFBR2 participates in TGF-β1 and miR-20b-5p regulation of E2F1 expression, we knocked down TGFBR2 in PC3 and DU145 cells and measured the effect of TGF-β1 and miR-20b-5p on E2F1 expression. qRT-PCR analysis showed that although TGF-β1 treatment significantly down-regulated the expression level of E2F1 mRNA in si-control-transfected cells, TGFBR2 knockdown eliminated the inhibitory effect of TGF-β1 on the expression of E2F1 (Figure 5A and Supplementary Figure 8). Consistent with the results of TGFBR2 knockdown, transfection of PC3 and DU145 cells with the miR-20b-5p mimic also abolished the inhibitory effect of TGF-β1 on E2F1 expression (Figure 5B). These findings indicate that TGFBR2 lies functionally upstream of E2F1 in the TGF-β1 signaling pathway.

Previous research has shown that Smad2/3 and E2F1 are critical mediators of the effects of TGF-β in both normal and cancer cells (18). For this reason, we treated PC3 cells with TGF-β1 and examined the effect of TGF-β1 and miR-20b-5p on Smad2 phosphorylation and EMT-related gene expression. The results showed that TGF-β1 markedly induced Smad2 phosphorylation and up-regulated the expression level of TGFBR2 and vimentin proteins but reduced E-cadherin expression (Figure 5C, lanes 2 and 3 vs. lane 1), suggesting that TGFBR2/Smad2 signaling mediates TGF-β1-induced EMT in PCa cells. However, miR-20b-5p mimic transfection could effectively suppress EMT in PC3 cells by down-regulating TGFBR2 expression and decreasing Smad2 activation, as evidenced by the increased expression of E-cadherin and decreased expression of vimentin (Figure 5C and Supplementary Figure 9). In order to determine the function of miR-20b-5p *in vivo*, we established PCa xenograft models by implanting PC3 cells overexpressing miR-20b-5p into nude mice. The animal study found that the tumor volumes were significantly decreased in nude mice implanted with miR-20b-5p-overexpressed PC3 cells compared with those in the control (Figures 5D,E). Western blot analysis also showed that the overexpression of miR-20b-5p in xenograft tissues increased phosphorylated Smad2 and E-cadherin, whereas it reduced TGFBR2 and vimentin protein level (Figure 5F). These results suggest that miR-20b-5p, TGFBR2, and E2F1 form a regulatory loop to regulate EMT induced by TGF-β1 in PCa cells (Figure 5G).

## Discussion

In this study, we found that the level of miR-20b-5p expression was lower in PC3, DU145, and 22RV1 cells than that in prostate epithelial cells, and TGF-β1 treatment further reduced the miR-20b-5p expression level. These findings suggest that miR-20b-5p inhibited TGF-β1-induced EMT in PCa cells and that miR-20b-5p down-regulation might facilitate EMT in PCa. The aim of the present study was to determine whether miR-20b-5p participates in TGF-β1-induced EMT in PCa cells and the mechanisms underlying this.

Previous studies have shown that miR-20a-5p and miR-20b-5p are transcribed from miR-17~92 and miR-106a~363 clusters, respectively, and these two miRNAs promote myoblast differentiation and repress myoblast proliferation by directly binding the 3′-UTR of E2F1 mRNA (19). miR-20b-5p has been confirmed to act as a tumor suppressor in renal cell carcinoma and bladder cancer (12, 13). These findings suggest that miR-20b-5p is involved in tumorigenesis and tumor development. In this study, we found that the expression of miR-20b-5p was down-regulated in PCa tissues, compared with that in BPH tissues. Aside from the expression of miR-20b-5p in PCa tissues, Hoey et al. recently reported that circulating miR-20b-5p is one of the non-invasive biomarkers that predict aggressive PCa after radical prostatectomy and that a high expression of miRNAs indicates a faster biochemical recurrence time (33). Due to the longer survival of PCa patients, we have not yet collected sufficient evidence to prove the relationship between miR-20b-5p expression and PCa prognosis. We identified TGFBR2 as a direct target of miR-20b-5p in PC3 and DU145 cells and found that miR-20b-5p negatively regulated TGFBR2 expression by targeting its 3′-UTR in PCa cells. Since it has been demonstrated that Smad2/3 and E2F1 are critical mediators of TGF-β-induced EMT in both normal and cancer cells (18), we examined the effects of TGF-β1 and miR-20b-5p on Smad2 phosphorylation and E2F1 expression. Our findings showed that TGF-β1 induced Smad2 phosphorylation and down-regulated E2F1 expression, with increased vimentin expression and reduced E-cadherin levels, that is, EMT. Others and our results have shown that miR-20b-5p can regulate cellular EMT (14). However, how miR-20b-5p regulates EMT marker genes and cell migration remains to be further studied.

The transcription factor E2F1 is involved in the cell cycle and in proliferation, apoptosis, and differentiation and can act as a tumor suppressor or oncogene (34–37). A recent study showed that E2F1 is closely associated with EMT in small cell lung cancer (32). Previous research has shown that TGF-β induces the post-translational protein expression of E2F1 (38). However, our findings showed that treating PC3 and DU145 cells with TGF-β1 reduced the levels of E2F1, mRNA, and protein expression at 24 h after TGF-β1 treatment. These results suggest that the TGF-β-E2F1 signaling axis plays a crucial role in regulating the tumor-suppressive effects and tumor-promoting effects of TGF-β.

A recent study indicated an important role in myoblast proliferation and differentiation through auto-regulation between E2F1 and miR-20a-5p/20b-5p (19). However, the existence of the E2F1-miR-20b-5p auto-regulatory feedback loop, and the importance of the role it plays in EMT in PCa cells, was unclear. Previous studies have found that the auto-regulatory feedback loops play an important role in regulating cell EMT (39–41). Our findings clearly showed that the E2F1-miR-20b-5p auto-regulatory feedback loop not only exists in PCa cells but also exerts a crucial role in EMT in PCa cells.

The transcription factor E2F1 is crucial for melanoma progression through directly transactivating the GABRE gene that expresses miR-224/miR-452. In addition, miR-224/452-mediated down-regulation of thioredoxin-interacting protein (TXNIP, also known as VDUP-1 or TBP-2) is essential for E2F1-induced EMT and invasion (42). Similar to previous findings, our results indicated that TGFBR2 is the direct target of miR-20b-5p in PCa cells. Notably, TGFBR2 and E2F1 formed a regulatory axis to modulate EMT induced by TGF-β1. In PCa xenograft models, the tumor volumes were significantly decreased compared with those in the corresponding control group. These results suggest that the miR-20b-5p/TGFBR2/E2F1 regulatory axis is involved in PCa development and progression through regulating EMT-related gene expression and that dysregulation of this regulatory axis contributes to EMT in PCa.

A number of recent studies have reported on the progression of PCa and EMT in TGF-β1 regulating PCa cells (43–46). However, in the present study, we found that the auto-regulatory axis miR-20b-5p/TGFBR2/E2F1, which is regulated by TGF-β1, is involved in PCa development and progression through regulating EMT-related gene expression. Targeting this newly identified regulatory axis provides a potential therapeutic strategy for aggressive PCa.

## Data Availability Statement

All datasets generated for this study are included in the article/Supplementary Material.

## Ethics Statement

The animal study was reviewed and approved by Institutional Animal Care and Use Committee of Hebei Medical University (approval ID: HebMU 20080026).

## Author Contributions

J-CQ designed and performed the experiments and wrote the manuscript. ZY, Y-PZ, B-SL, Y-WY, K-LL, W-YX, and C-BQ contributed to experimental work and data analysis. C-BQ and WL conducted the experiments and revised the manuscript. All authors have read and approved the final manuscript.

### Conflict of Interest

The authors declare that the research was conducted in the absence of any commercial or financial relationships that could be construed as a potential conflict of interest.
