# Supplementary material for: miR-20b-5p, TGFBR2, and E2F1 Form a Regulatory Loop to Participate in Epithelial to Mesenchymal Transition in Prostate Cancer
Source: Front Oncol. 2020 Jan 15;9:1535. doi: 10.3389/fonc.2019.01535 (PMC6974577; doi:10.3389/fonc.2019.01535)
Supplement: Supplementary file 1 [file Data_Sheet_1.PDF]

Supplementary material

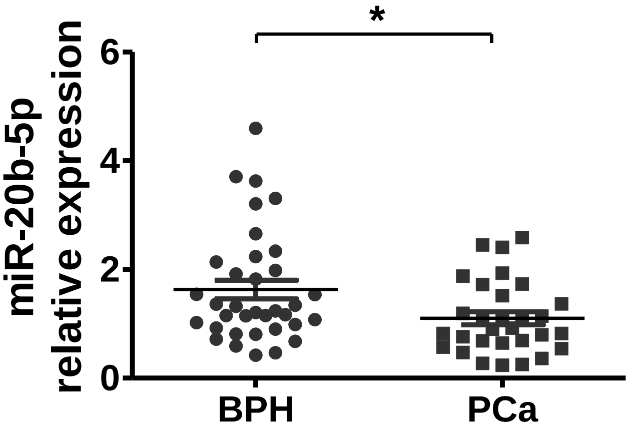

Supplementary figure S1. RT-PCR detected the expression of miR-20b-5p in PCa (n = 30) and benign prostatic hyperplasia (BPH, n = 35). *P<0.05 vs. BPH

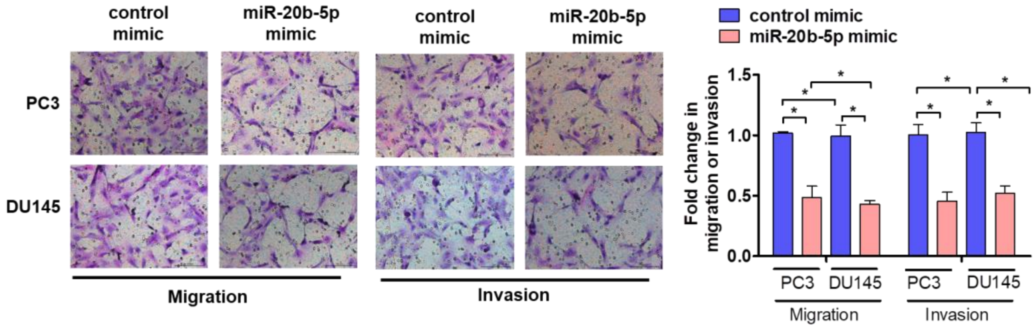

Supplementary figure s2. PC3 and DU145 cells were transfected with miR-20b-5p mimic and control mimic, and then transwell was used to detect migration and invasion. *P<0.05 vs. corresponding control.

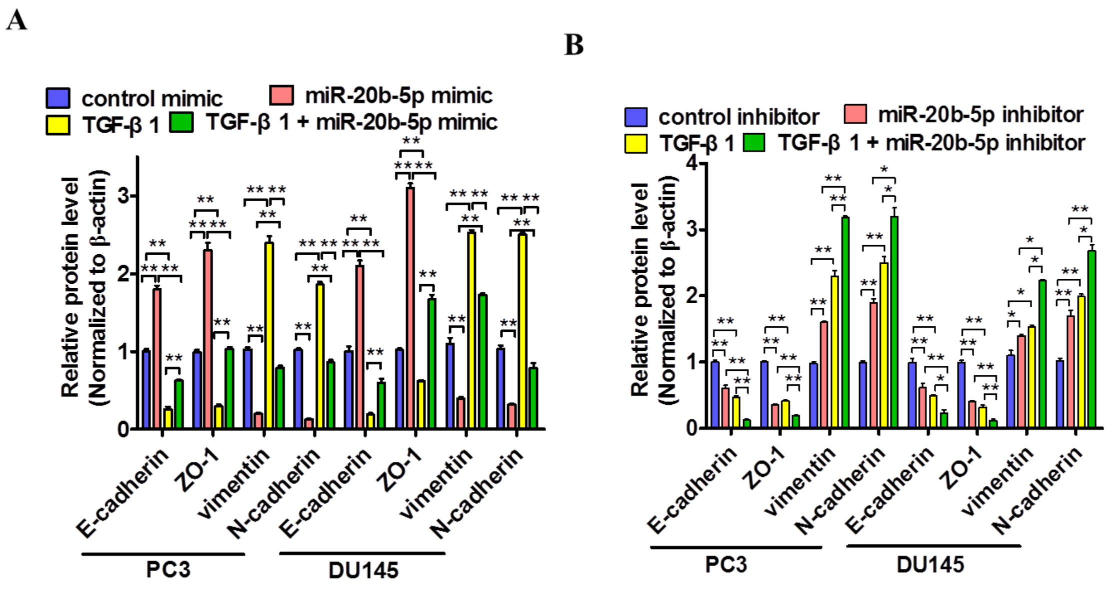

Supplementary figure s3. A and B, Quantitative analysis of Figure 2D and E. Expression of indicated proteins ware quantitated by densitometry, and values were normalized to total β–actin. n=3. *P<0.05, **P<0.01 vs. corresponding control.

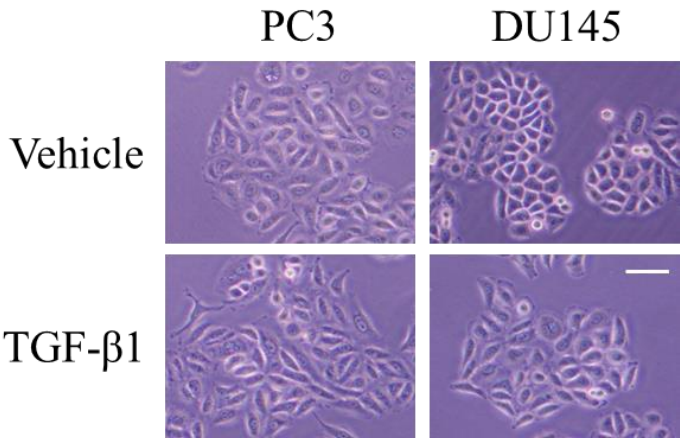

Supplementary figure s4. Phase-contrast microscope observed cell morphology of PC3 and DU145 treated with or without TGF-β1. Scale bars=25 μm.

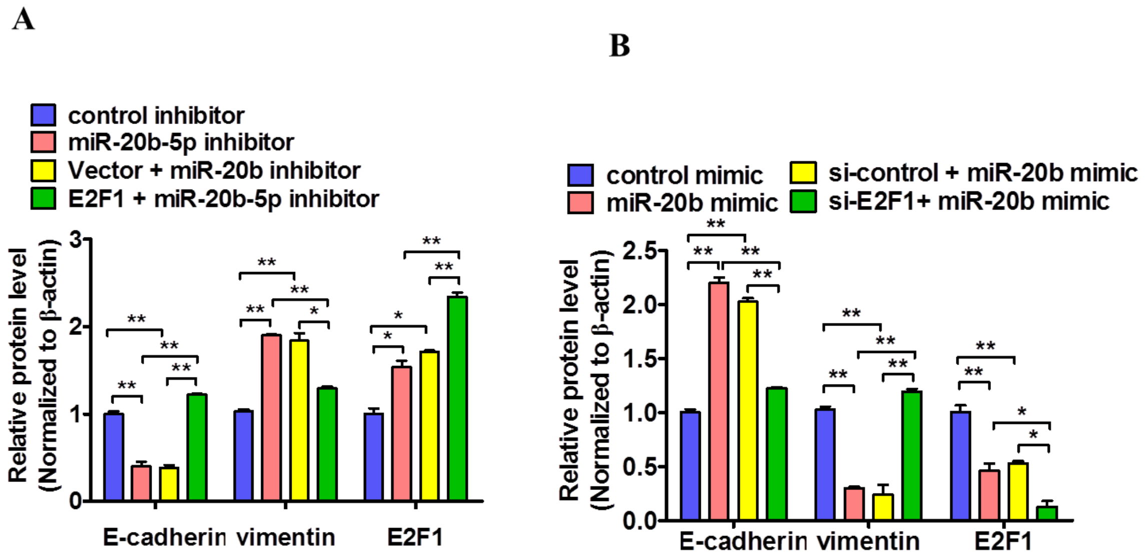

Supplementary figure s5. A and B, Quantitative analysis of Figure 3E and F. Expression of indicated proteins ware quantitated by densitometry, and values were normalized to total β–actin. n=3. *P<0.05, **P<0.01 vs. corresponding control.

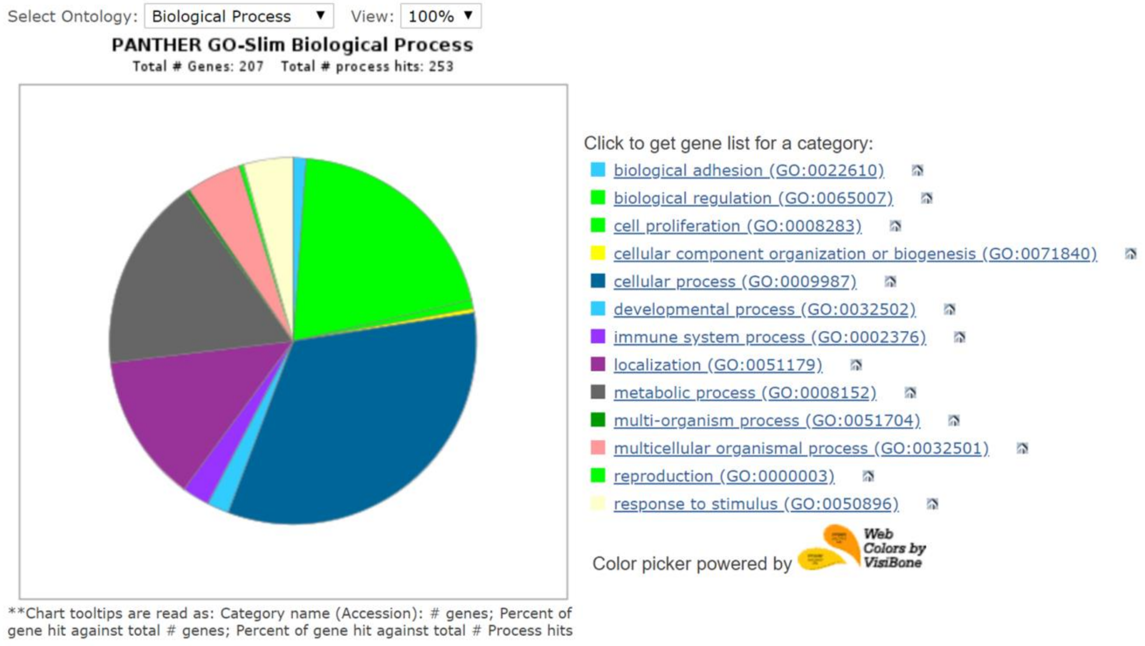

Supplementary figure s6. Prediction of miR-20b-5p targets that involved in biological adhesion in the function of biological process.

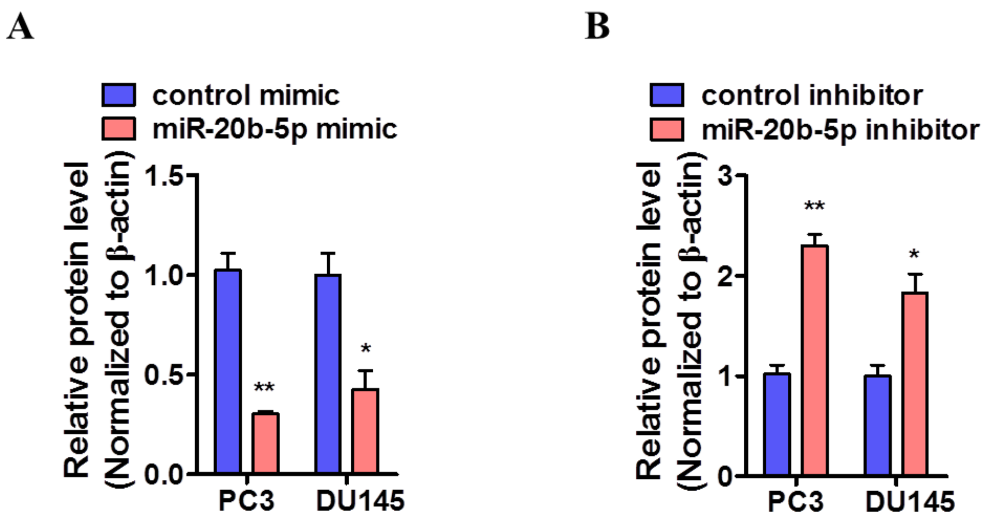

Supplementary figure s7. A and B, Quantitative analysis of Figure 4G and H. Expression of indicated proteins ware quantitated by densitometry, and values were normalized to total β–actin. n=3. *P<0.05, **P<0.01 vs. corresponding control.

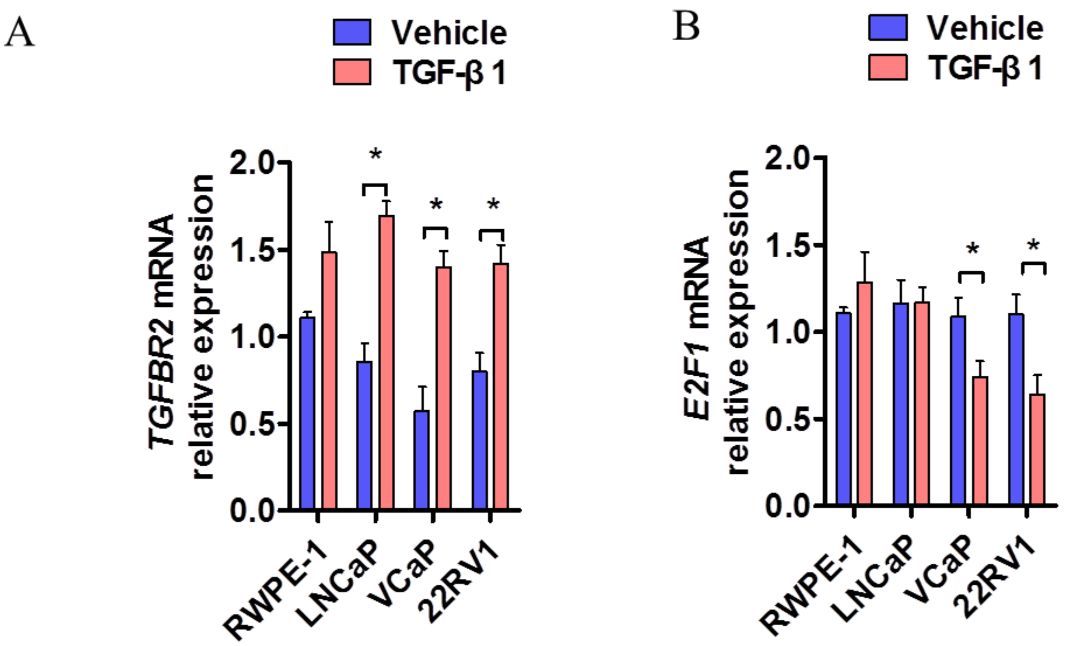

Supplementary figure s8. A and B, TGFBR2 (A) and E2F1 (B) mRNA was detected by qRT-PCR in TGF-β1-treated RWPE-1,LNCaP,VCaP and 22RV1 cells for 48 h. *P < 0.05 versus vehicle.

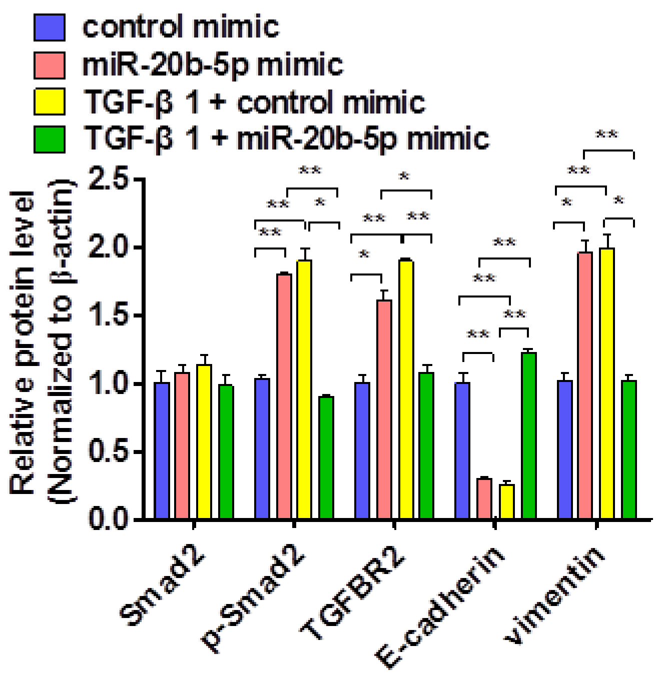

Supplementary figure s9. Quantitative analysis of Figure 5C. Expression of indicated proteins ware quantitated by densitometry, and values were normalized to total β–actin. n=3. *P<0.05, **P<0.01 vs. corresponding control.
